# Supplementary material for: Robust polyimide nano/microfibre aerogels welded by solvent-vapour for environmental applications
Source: R Soc Open Sci. 2019 Aug 7;6(8):190596. doi: 10.1098/rsos.190596 (PMC6731741; doi:10.1098/rsos.190596)
Supplement: The schematic diagram of Automated Filter Tester, photographs of PI-N/MFA0 and reversible manual compression of PI-N/MFAs [file rsos190596supp2.doc]

**Supplemental Material**

**Robust Polyimide Nano/Microfiber Aerogels Welded by Solvent-Vapor for Environmental Applications**

Ying Shen ^a^, Dawei Li ^a^, Bingyao Deng *^a^, Qingsheng Liu ^a^, Huizhong Liu ^a^ and Tong Wu ^b^

1. Key Laboratory of Eco-Textiles (Ministry of Education), Nonwoven Technology Laboratory, Jiangnan University, Wuxi 214122, China.
2. The Wallace H. Coulter Department of Biomedical Engineering, Georgia Institute of Technology and Emory University, Atlanta, Georgia 30332, United States

^*^Corresponding author: bydeng@jiangnan.edu.cn

Supplementary material Contains:

Supplementary Figures S1-S3;

Movie S1.

| 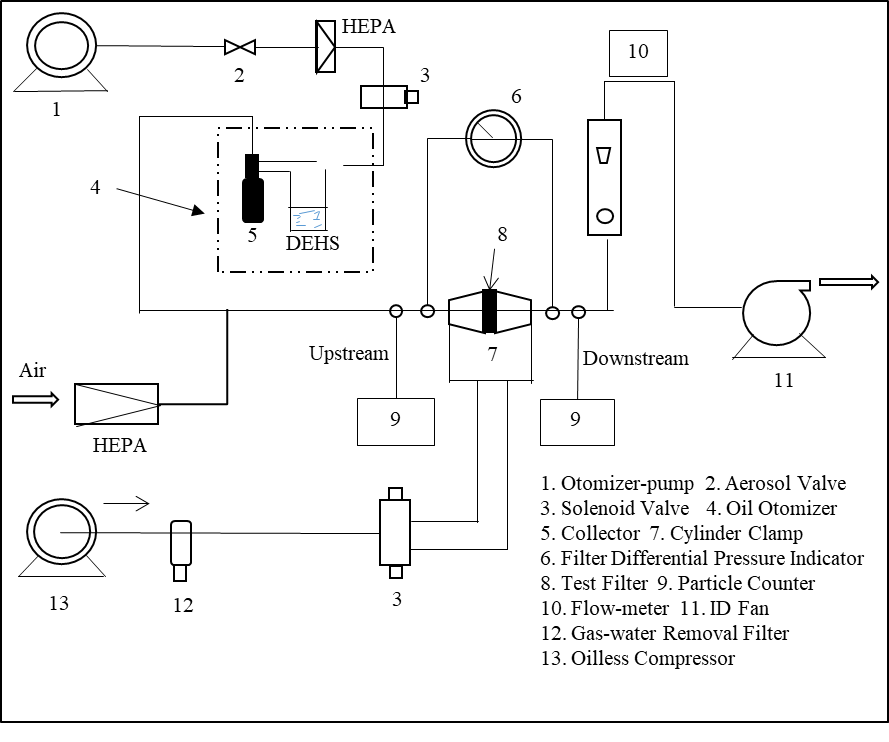 |
| --- |
| Fig. S1. Experimental set-up for testing filtration efficiency. |

This machine was used to evaluate the filtration efficiency and air permeability of PI nanofiber aerogel composite filters (PI-NFACFs). It was designed based on the standard test of GB2626-2006 and EN1822-3:1998. As shown in Fig. S1, it consisted of a solid NaCl aerosol generation unit, a filter holder, a pair of laser particle counters, and a combination of a flow meter and two electronic pressure transducers. The generated NaCl aerosols have mean particle diameters ranging from 0.3 ~ 10 μm. When measuring, the NaCl aerosols were fed into the filter holder with an inner area of 100 cm^2^ and drawn through the filter with a continuous airflow rate of 84 L/min.

| 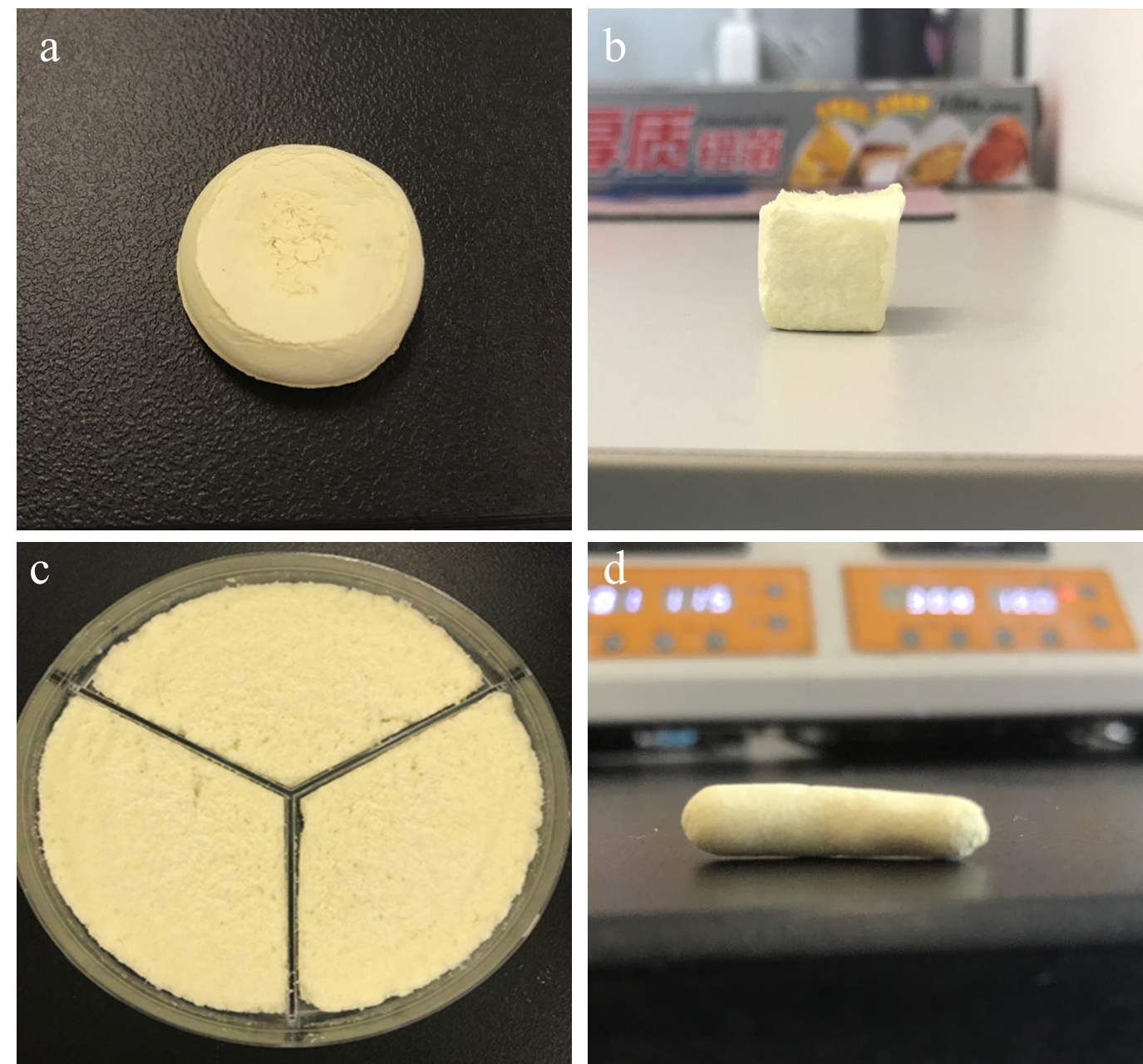 |
| --- |
| Fig. S2. Photographs of PI-N/MFA_0_ prepared from different molds. |

As illustrated in Fig. S2, by using different molds, PI-N/MFA_0_ products with various shapes could be readily obtained, such as cylinder, cube, sector and long strip shape.

| 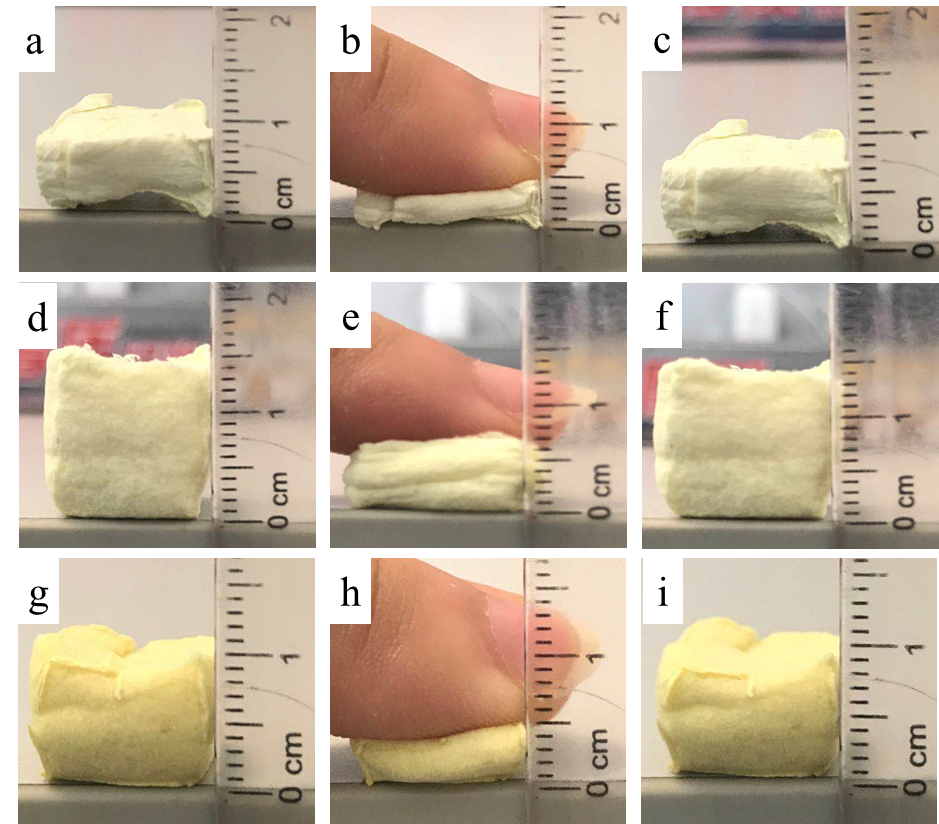 |
| --- |
| Fig. S3 Reversible manual compression, a-c: PI-N/MFA_0_; d-f: PI-N/MFA_50_; g-i: PI-N/MFA_100_. |

Due to the bonding of fibers in the skeletons resulting from both physical entanglement and vapor-induced welding, the PI nano/microfiber aerogels (PI-N/MFAs) exhibited extraordinary flexibility and toughness. As shown in Fig. S3, a monolith of PI-N/MFAs could recover to its initial dimensions immediately without any fracture when crushed by fingers, demonstrating the good elastic resilience of the PI-N/MFAs.

Movie for supporting information

|  |
| --- |
| . The dynamic compressive behavior of the PI-N/MFA_50_. |

Movie S1 showing dynamic compressive behavior of the PI-N/MFA_50_. It was indicated that the PI-N/MFA_50_ possessed great compressive resilient performance.
